# *Horse to human: Streptococcus equi* septicemia presenting as endogenous endophthalmitis

**DOI:** 10.1016/j.ajoc.2023.101974

**Published:** 2023-12-08

**Authors:** Robert E. Morris, Sean Doherty, Matthew H. Oltmanns, Mathew R. Sapp, Kevin Wells, Hershel R. Patel

**Affiliations:** a Retina Specialists of Alabama, LLC, Birmingham, AL, USA; b Helen Keller Foundation for Research and Education, Birmingham, AL, USA; c University of Alabama at Birmingham (UAB), Department of Ophthalmology, Birmingham, AL, USA; d University of Massachusetts Chan Medical School, Worcester, MA, USA; e The Retina Center PA, Bryan, TX, USA; f Centers for Retina & Macular Disease, Winter Haven, FL, USA

**Keywords:** Endogenous endophthalmitis, Vitrectomy, Streptococcus equi, Septicemia

## Abstract

**Purpose:**

To present a rarely reported systemic infection with *streptococcus equi subspecies zooepidemicus* (*streptococcus equi*), transmitted from a horse, and to describe successful treatment when complicated by endogenous endophthalmitis.

**Observations:**

We diagnosed suspected *streptococcus equi* septicemia presenting as loss of vision in the right eye of an otherwise healthy polo player/horse trainer. He received immediate intravenous antibiotics and three vitrectomies with two intravitreal antibiotic injections during the first week, to cure infection and subsequent retinal detachment. Blood and initial vitreous cultures rapidly grew *streptococcus equi.* The septicemia was quickly controlled by systemic antibiotics without developing commonly seen and often fatal meningitis. The right eye recovered 20/30 visual acuity three months post infection.

**Conclusions:**

Presentation of this rare septicemia as endogenous endophthalmitis illustrates the potentially lifesaving role of early diagnosis by the ophthalmologist. Immediate and recurrent vitrectomy in conjunction with intravitreal and systemic antibiotic therapy resulted in recovery of near normal vision, whereas less timely and interventional treatments have failed heretofore.

## 1 Introduction

*Streptococcus equi subspecies zooepidemicus (streptococcus equi)* is a group c streptococcus that is rarely transmitted from horses to humans, and typically accounts for less than 1 % of all cases of bacteremia in humans.^1^, ^2^, ^3^ Streptococcus equi is usually transmitted through contact with the nasal discharge of an infected horse. Human infection is associated with close contact with horses and consumption of unpasteurized dairy products.^2^^,^^4^ A commensal organism in horses, in humans it typically causes fulminant septicemia with severe complications, most notably meningitis, with a mortality rate of over 20 %.^1^

After a literature review on October 21, 2023, using search engines PubMed and Google Scholar and key words “*streptococcus equi*” and “endogenous endophthalmitis,” we found only three cases of endogenous endophthalmitis secondary to *streptococcus equi* septicemia.^3^^,^^5^^,^^6^ None of these case reports described vision loss as the primary presenting symptom, and no treated eye regained useful vision.

We present a case of *streptococcus equi* septicemia with a primary presenting symptom of vision loss. Endogenous endophthalmitis from *streptococcus equi* septicemia was promptly diagnosed and treated, forestalling-life threatening sequalae and leading to successful recovery of vision.

## 2 Case report

A 48-year-old man, previously in good health, came to our retina clinic in no acute distress, after experiencing four days of spontaneous, painless loss of vision in the right eye. He had no history of ocular trauma, operative procedure, or prior eye disease. On the second day of symptoms, two days prior to presentation to our clinic, the patient experienced a marked decrease in vision, and he was seen by an ophthalmologist who initiated a diagnostic work up for sterile uveitis and began treatment with topical cycloplegics and steroids. Of note, the patient reported that he lived on a farm where he cared for polo horses and was himself a polo player.

**Fig. 1:**
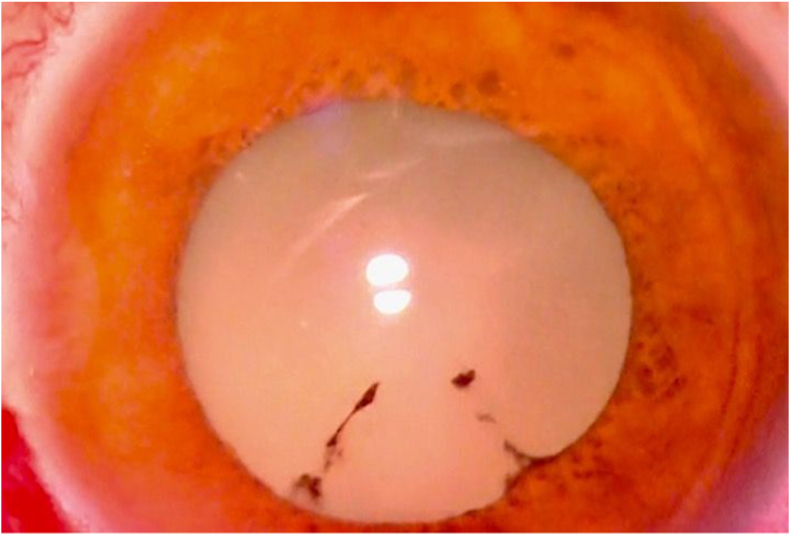
Anterior segment at presentation, with purulence covering the entire posterior lens surface, obliterating all fundus view.

On examination, the right eye had only light perception visual acuity. There was no conjunctival injection, a clear cornea, modest cell and flare, and a 2 mm hypopyon. Purulent vitreous infiltrate was visible abutting the entire posterior capsule of the clear crystalline lens, with a 9 mm pupil already pharmacologically dilated (Fig. 1), with no red reflex. Ultrasonography revealed homogeneous, modest strength echoes throughout the entire vitreous with no posterior vitreous detachment (PVD) or retinal detachment. The left eye was normal to examination. Complete vitreous purulence was interpreted to be incompatible with a sterile uveitis. Upon further questioning, the patient admitted to a slight fever and a sore right elbow, right wrist, and left knee. Septicemia with right eye endogenous endophthalmitis was suspected.

The patient was immediately moved to an adjacent emergency room while infectious disease consultation was obtained, leading to a tentative clinical diagnosis of *streptococcus equi* septicemia*.* Intravenous vancomycin was begun immediately after blood culturing, and vitrectomy was promptly performed (KW) with intravitreal injection of 1.0 mg of vancomycin and 2.25 mg of ceftazidime (Video). Due to limited visualization, no periretinal manipulations were performed, leaving a thin layer of purulent cortical vitreous.

The following day the patient had lavage of purulence from the three symptomatic joints with drains left in place for two days, and systemic antibiotic therapy was continued with ceftriaxone. *Streptococcus equi* *subspecies zooepidemicus* quickly grew from cultures of blood, the right vitreous, all three affected joints, and from urine. Systemic treatment with ceftriaxone continued throughout a nine-day hospitalization, and cefazoline was continued twice daily after discharge.

During hospitalization, due to persistent and recurrent vitreous purulence, a second vitrectomy with 1.0 mg of vancomycin intravitreal injection was performed (MO) on day three. A superonasal site of infection breakthrough from the choroid, and a surrounding rhegmatogenous retinal detachment with partial PVD, were then discovered. On hospital day five, with the endophthalmitis now seemingly controlled, a third vitrectomy was performed (MS) to repair the extramacular retinal detachment, and silicone oil was placed. Six weeks after discharge, vitrectomy with silicone oil removal and cataract extraction with placement of an intraocular lens were performed (RM).

**Fig. 2:**
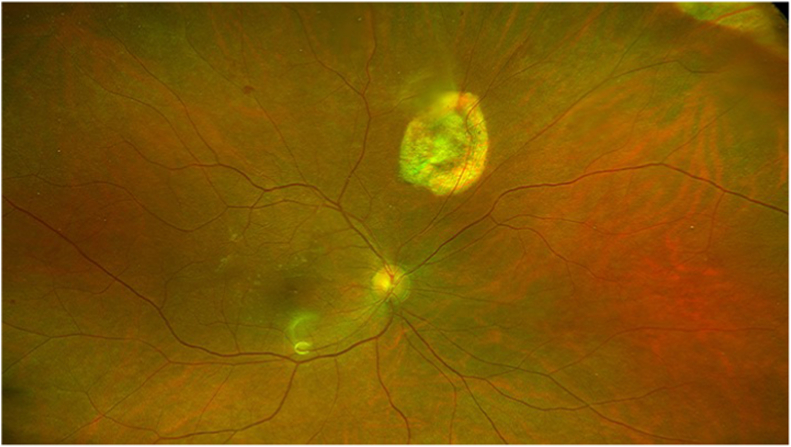
Wide angle image of the retina three months postoperatively, showing the site of original choroidal infection superonasal. Reflection overlying the inferior macula is artifactual.

**Fig. 3:**
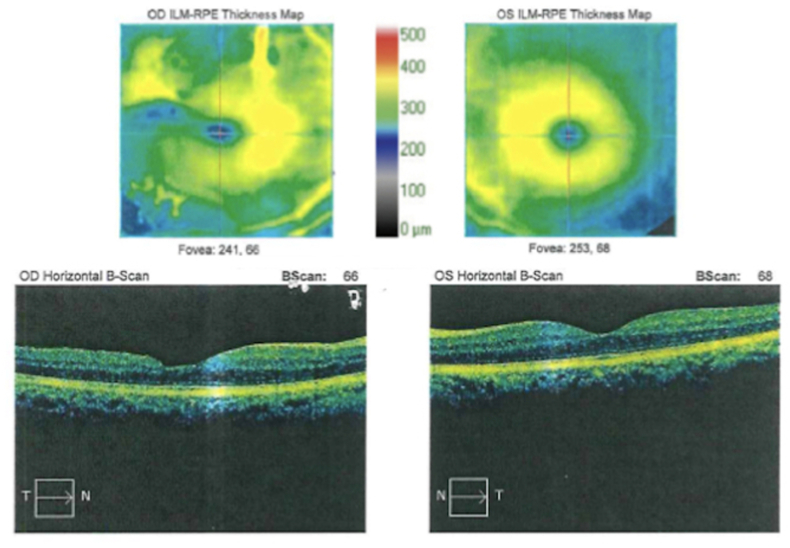
Optical coherence tomography (OCT) image of right macula three years post treatment, with visual acuity of 20/30 and moderate retinal atrophy temporally (endophthalmitis retinopathy). Unaffected left macula included for comparison.

The patient recovered 20/30 corrected visual acuity in the right eye three months post infection, with a stable retina (Fig. 2). Three years post infection the right eye maintained 20/30 visual acuity with a stable retina and macula (Fig. 3), and a scar at the site of choroidal breakthrough superonasal.

## 3 Discussion

The first known *case of streptococcus equi* endogenous endophthalmitis was mentioned in a brief 1995 French report which focused primarily on meningitis and the systemic infection, with no mention of visual outcome.^5^ In 2009, a second *streptococcus equi* endogenous endophthalmitis case was described in which a patient was admitted for fever and hypotension of unknown origin.^3^ Intravitreal antibiotic injections were performed on the second day of hospitalization, the first day of bilateral vision loss. However, vitrectomy was not performed until the eleventh hospital day, and bilateral blindness resulted from endophthalmitis retinopathy with retinal detachment. A third case was reported in 2015 with meningitis as the presenting feature.^6^ Left eye vision loss was noted on the second day of hospitalization and vitrectomy was “eventually” performed with “markedly impaired visual acuity” in the affected eye reported as the final outcome.

The current case illustrates the life-saving potential of early recognition by an ophthalmologist, with immediate infectious disease consultation. Such recognition led to early diagnosis and treatment, preventing often-fatal meningitis and systemic deterioration that otherwise may have precluded successful treatment of concomitant streptococcus equi endophthalmitis by recurrent vitrectomy.

While the patient's systemic involvement was recognized relatively early, the endophthalmitis was already advanced on the fourth day of symptoms and was suspected to be caused by a virulent bacterium. To limit endophthalmitis retinopathy, our goals were to restore fundus visualization and to sterilize the vitreous cavity as quickly as possible.^7^^,^^8^ This required both removal of infected tissue by vitrectomy and sustained intravitreal antibiotic levels.

The treatment plan in this case was similar to the approach we have employed successfully in the management of exogenous endophthalmitis following cataract surgery. Using fundus obscuration as the indication and vitrectomy as the predominate initial treatment, we previously reported an increase in salvage of 20/40 visual acuity to 79 % of cases, versus 56 % reported using the less interventional Endophthalmitis Vitrectomy Study (EVS) protocol from 1995.^7^, ^8^, ^9^ We also cited atrophic defects from endophthalmitis retinopathy as the leading source of retinal detachment in exogenous endophthalmitis, as occurred at the site of infection breakthrough from the choroid in this case of endogenous endophthalmitis (Fig. 2).

The orthopedic treatment of this patient's infected knee, elbow and wrist consisted of initial lavage and placement of drains until purulence was resolved, consistent with the general concepts of concomitant abscess drainage and antibiotic therapy. Similarly, we urge ophthalmologists to consider the importance of early and recurrent purulence removal and antibiotic injection to prevent retinopathy, the primary cause of visual loss in endophthalmitis.^7^^,^^9^

## 4 Conclusions

Despite improvements in medical and surgical management of endogenous endophthalmitis, visual outcomes remain poor. In a 2014 review of 342 endogenous bacterial endophthalmitis cases, Jackson et al. found that more than half of eyes retained less than 20/200 vision, and a quarter of eyes were enucleated/eviscerated.^10^ Importantly, only 20 % of eyes were treated with vitrectomy. This case report suggests that more frequent and timely vitrectomy may improve endogenous bacterial endophthalmitis outcomes.^7^^,^^8^

## Patient Consent

The involved patient gave written, informed consent to publication of this case report.

## Authorship

All authors attest that they meet the current ICMJE criteria for authorship.

## Funding

Helen Keller Foundation for Research & Education through grants from the Hanna Charitable Trust. The funding organizations had no role in the design or conduct of this research.

## Intellectual property

We confirm that we have given due consideration to the protection of intellectual property associated with this work and that there are no impediments to publication, including the timing of publication, with respect to intellectual property. In so doing we confirm that we have followed the regulations of our institutions concerning intellectual property.

## Research ethics

We further confirm that any aspect of the work covered in this manuscript that has involved human patients has been conducted with the ethical approval of all relevant bodies and that such approvals are acknowledged within the manuscript.

IRB approval was obtained (required for studies and series of 3 or more cases)

Written consent to publish potentially identifying information, such as details or the case and photographs, was obtained from the patient(s) or their legal guardian(s).

## Authorship

The International Committee of Medical Journal Editors (ICMJE) recommends that authorship be based on the following four criteria:

1. Substantial contributions to the conception or design of the work; or the acquisition, analysis, or interpretation of data for the work; AND
2. Drafting the work or revising it critically for important intellectual content; AND
3. Final approval of the version to be published; AND
4. Agreement to be accountable for all aspects of the work in ensuring that questions related to the accuracy or integrity of any part of the work are appropriately investigated and resolved.

All those designated as authors should meet all four criteria for authorship, and all who meet the four criteria should be identified as authors. For more information on authorship, please see http://www.icmje.org/recommendations/browse/roles-andresponsibilities/defining-the-role-of-authors-and-contributors.html#two.

All listed authors meet the ICMJE criteria. We attest that all authors contributed significantly to the creation of this manuscript, each having fulfilled criteria as established by the ICMJE.

One or more listed authors do(es) not meet the ICMJE criteria.

We believe these individuals should be listed as authors because:

[Please elaborate below]  

We confirm that the manuscript has been read and approved by all named authors.

We confirm that the order of authors listed in the manuscript has been approved by all named authors.

## Contact with the editorial office

The Corresponding Author declared on the title page of the manuscript is: Robert E. Morris, MD.

This author submitted this manuscript using his/her account in EVISE.

We understand that this Corresponding Author is the sole contact for the Editorial process (including EVISE and direct communications with the office). He/she is responsible for communicating with the other authors about progress, submissions of revisions and final approval of proofs.

We confirm that the email address shown below is accessible by the Corresponding Author, is the address to which Corresponding Author's EVISE account is linked, and has been configured to accept email from the editorial office of American Journal of Ophthalmology Case Reports:

rmorris@rmeyes.com.

Someone other than the Corresponding Author declared above submitted this manuscript from his/her account in EVISE:

Sean Doherty.

281 Lincoln St.

Worcester, MA, 01605 Sean.doherty@umassmed.edu.

+1(774)219 4358 cell.

We understand that this author is the sole contact for the Editorial process (including EVISE and direct communications with the office). He/she is responsible for communicating with the other authors, including the Corresponding Author, about progress, submissions of revisions and final approval of proofs.

Patient Consent for Publication of Material in American Journal of Ophthalmology Case Reports.

The following information must be completed in order for this form to be processed accurately.

Streptococcus Equi Septicemia Presenting as Endogenous Endophthalmitis: Rare Salvage of Functional Vision Author(s) name(s) (type):

## Patient or representative to fill in items below

I hereby give my consent for images or other clinical information relating to my case to be reported in the *American Journal of Ophthalmology Case Reports (AJO Case Reports).*
I understand that my name, initials, or any protected health information such as my identification number, billing information, address, etc. will not be published and that every effort will be made to conceal my identity, but that anonymity cannot be guaranteed. Identifying (e.g., face photographs) and/or non-identifying (e.g., diagnostic images) images may be published.
I understand that the material may be published in the *AJO Case Reports,* on its Web site, and in products derived from the *AJO Case Reports.* As a result, I understand that the material may be seen by the general public.
I declare, in consequence of granting this permission, that I have no claim on ground of breach of confidence or any other ground in any legal system against the author(s) and their agents, publishers, successors and assigns in respect of such use of the photograph(s) and such materials.
I agree to release and discharge the author(s) and any editors or other contributors and their agents, publishers, successors and assigns from any and all claims, demands or causes of action that I may now have or may hereafter have for libel, defamation, invasion of privacy, copyright or moral rights or violation of any other rights arising out of or relating to any use of my image or case history.

## CRediT authorship contribution statement

**Robert E. Morris:** Conceptualization, Methodology, Writing – review & editing, Supervision, Funding acquisition. **Sean Doherty:** Writing – original draft, Review & Editing, Visualization, Project administration. **Matthew H. Oltmanns:** Investigation. **Mathew R. Sapp:** Investigation. **Kevin Wells:** Investigation. **Hershel R. Patel:** Investigation.

## Declaration of competing interest

The authors declare that they have no known competing financial interests or personal relationships that could have appeared to influence the work reported in this paper.
